# Sweet taste in apple: the role of sorbitol, individual sugars, organic acids and volatile compounds

**DOI:** 10.1038/srep44950

**Published:** 2017-03-21

**Authors:** Eugenio Aprea, Mathilde Charles, Isabella Endrizzi, Maria Laura Corollaro, Emanuela Betta, Franco Biasioli, Flavia Gasperi

**Affiliations:** 1 Research and Innovation Centre, Fondazione Edmund Mach, Via E. Mach 1, 38010 San Michele all’Adige, Italy

## Abstract

Sweetness is one of the main drivers of consumer preference, and thus is given high priority in apple breeding programmes. Due to the complexity of sweetness evaluation, soluble solid content (SSC) is commonly used as an estimation of this trait. Nevertheless, it has been demonstrated that SSC and sweet taste are poorly correlated. Though individual sugar content may vary greatly between and within apple cultivars, no previous study has tried to investigate the relationship between the amount of individual sugars, or ratios of these, and apple sweetness. In this work, we quantified the major sugars (sucrose, glucose, fructose, xylose) and sorbitol and explored their influence on perceived sweetness in apple; we also related this to malic acid content, SSC and volatile compounds. Our data confirmed that the correlation between sweetness and SSC is weak. We found that sorbitol content correlates (similarly to SSC) with perceived sweetness better than any other single sugar or total sugar content. The single sugars show no differentiable importance in determining apple sweetness. Our predictive model based on partial least squares regression shows that after sorbitol and SSC, the most important contribution to apple sweetness is provided by several volatile compounds, mainly esters and farnesene.

Modern plant-breeding methods, based on screening seedlings from breeding progenies with DNA-based markers associated with traits of interest (marker-assisted selection, MAS), offer efficient tools for cultivar improvement, especially for fruit trees with long juvenile periods^1^. Several quantitative trait loci (QTLs) for important fruit quality traits in apples, including volatile compounds^2^,^3^, phenolic compounds^4^, texture parameters^5^, shape and major physiological parameters^6^,^7^, have been identified for use in MAS. Sweetness and sourness have been recognized as important drivers of apple consumer preference^8^; recent studies have confirmed this^9^,^10^. For this reason, sweetness is one of the most important fruit sensory quality traits taken into account in breeding programmes^11^. The best evaluation and description of perceived quality of food can be provided only by trained panellists through sensory analysis. However, examples of the application of sensory techniques to apple quality evaluation are rare in literature, mainly because sensory methodologies require more time and specialized resources than do instrumental characterizations^12^. For instance, Corollaro *et al*., reported a detailed and comprehensive methodology for sensory profiling of apples based on a consensus vocabulary^13^ and studied the link between sensory attributes and instrumental data^14^. Nevertheless, the inclusion of sensory descriptive analysis in breeding programmes is often too expensive and not suited to large numbers of samples. Moreover, the limited number of fruits produced in breeding programmes may be too few, or may be unsuitable for a full sensory profile. For these reasons, the evaluation of quality traits is mostly based on easily measurable analytical features that are indirectly related to the desired quality traits.

The soluble solid content (SSC) expressed as °Brix is commonly used as an estimate of fruit sweetness and is included in assessments of the postharvest quality of apples^11^,^15^. However, several studies showed that prediction of sweetness by °Brix in apples is inadequate^14^,^16^. In general, sweetness prediction in apples is a difficult task^14^,^17^. Harker *et al*., found that SSC and sweet taste are poorly correlated, and recommended the use of trained sensory panels for the evaluation of sweetness in apple^16^. Sweetness perception is influenced by other sensory characteristics. For example, several authors have investigated the relationship between texture properties and sweetness. Harker *et al*., posited a relationship between perceived sweetness and the amount of juice release during breakdown of apple flesh, rather than measures of sugar and acid content^18^. Their results did not clearly support a direct relationship between juiciness and sweetness, and these researchers verified that perception of sweetness is not affected by the volume or rate of release of juice into the mouth during consumption^18^. Echeverría *et al*. reported that apples with high levels of mealiness are perceived as being less sweet, while samples with low mealiness are perceived as sweeter by panellists, although this effect was not supported by the correlation between the two factors (r = −0.15)^17^. The relationship between the colour of the apple flesh and sweetness or sourness was reported as well. This relationship is likely due to an indirect correlation between changes in chemical composition during apple ripening and the consumer’s expectation of a sourer taste from a green-fleshed apple and vice versa^14^. This influence of colour on sweetness is a known phenomenon^19^.

The interaction between sweet sensation and odour is another well-documented phenomenon^20^,^21^. When a sipped solution is presented simultaneously with a “sweet-smelling” or “sweet-congruent” odorant, the solution is perceived to be sweeter. This phenomenon is known as odour-induced enhancement of taste perception^20^.

Studies of tomatoes and strawberries have highlighted the effect of volatile compounds on the perceived sweetness intensity of these fruits^22^,^23^.

However, the main elicitors of perceived sweetness in fruit are its sugars. The main sugars and sugar alcohols present in apples are sucrose, fructose, glucose, xylose and sorbitol^24^. While the ratios between individual sugars may vary greatly between and within apple cultivars, to the best of the authors’ knowledge no study has previously tried to investigate the relationship between apple sweetness and the amount of individual sugars, “sweet” polyalcohols (such as sorbitol) or ratios of these amounts. Neither has the possible influence of volatile compounds been tested. A plausible hypothesis is that the ratio between single sugar content (mainly sucrose, glucose and fructose), sorbitol and organic acids may explain apple sweetness better than the ratio between total sugar and organic acid content.

In this study we evaluate: (i) the influence of total sugar, single sugar and sorbitol content on perceived sweetness in apples, in association with malic acid (the main organic acid in apple) and SSC; and (ii) the influence that volatile compounds may have on sweet taste perception in apple. To do this, single sugars (sucrose, glucose, fructose, xylose), sorbitol, malic acid, SSC and volatile compounds were measured in apples and compared with sweetness, as measured by a trained sensory panel, using the same fruits.

## Results and Discussion

### Sugar content

The amount of sugar present in analysed apples is reported in Table 1. The total amount of sugars ranges from 74.7 to 142.9 kg^−1^, with an average concentration of 116.8 g kg^−1^. In agreement with previous reports, sucrose (41.8%) and fructose (39.1%) are, with few exceptions, the most abundant sugars, followed by glucose (18.3%)^16^,^25^,^26^. The amount of sucrose, fructose and glucose ranges between 22.2 g kg^−1^ and 91.0 g kg^−1^, 27.0 g kg^−1^ and 61.0 g kg^−1^ and 11.1 g kg^−1^ and 30.2 g kg^−1^, respectively. Xylose, a major component of xyloglucans^27^, represents only a small fraction of the measured sugars (0.8%), ranging between non-detectable and 1.8 g kg^−1^. Similar levels have been reported in previous investigations^24^,^28^.

Sorbitol, the major sugar alcohol in apples^28^, was present in concentrations between 1.3 g kg^−1^ and 12.9 g kg^−1^. Sorbitol and glucose, formed from the products of photosynthesis in leaves, are the translocation sugars flowing through the phloem to reach fruit tissue, where they are converted, depending on the developmental stage, into fructose, glucose, malic acid, or starch^29^. Sorbitol is preferentially converted into fructose while glucose is preferentially incorporated into starch^30^. In apples, only a small fraction of fructose is incorporated into starch; fructose instead accumulates in the vacuoles of apple cells^30^. As a result, fructose is always higher than glucose in fruit tissue as confirmed by our data (Table 1).

### Soluble solids

Soluble solid content (SSC) in the analysed apples varied from 9.5 to 15.8 °Brix, with an average value of 13.2 °Brix (Table 1). SSC, a main parameter for assessing post-harvest apple quality^16^, generally increases during storage^31^.

### Organic acids

The content of malic acid, the main organic acid in apples that accounts for approximately 90% of the acid content^25^, varied from 12.9 to 78.6 g kg^−1^ (Table 1). Content generally decreases during ripening and storage of apples^25^. As the analysed apples were all harvested at commercial ripening and submitted to the same storage conditions, the high variability in malic acid content may be related both to variable ability to accumulate malate in apple parenchyma cells between the cultivars^32^ and to varying maturity at commercial ripening^33^.

The amount of ascorbic acid was below 10 mg kg^−1^ (quantification limit) for all the analysed samples, in agreement with a previous report^34^. These data are not further discussed.

### Effect of sugars, malic acid and soluble solids on perceived sweetness

The apples evaluated by the panel received a rating for sweetness between 27.3 and 69.2 on the 0–100 linear scale (Table 2). The correlations between sweetness intensity and individual sugars, total sugar and SSC are reported in Table 3. Sorbitol (the sole sugar alcohol measured) (r = 0.661; p < 0.001) and total sugars (r = 0.410; p = 0.009) show significant positive correlations with sweetness. Sorbitol can explain 44% of sweetness, whereas the total sugars account for only 17%. Malic acid is negatively correlated to sweetness (r = −0.449; p = 0.05). SSC is positively correlated to sweetness (r = 0.635; p < 0.001), similar to the correlation for sorbitol. Harker and co-workers reported a lower average correlation (r = 0.41) between SSC and sweetness when measured by a trained panel^16^, accounting for only 17% of sweetness, a correlation similar to that observed for total sugar in the present investigation. We also found that sorbitol is positively correlated to SSC (r = 0.514; p = 0.001), glucose (r = 0.437; p = 0.006), fructose (r = 0.486; p = 0.002) and total sugar content (r = 0.578; p < 0.001) (Table 3). Guan and co-workers reported a significant correlation (0.66) between SSC and sorbitol^11^. Our findings corroborate that SSC measurement can explain only a fraction of the perceived sweetness in apple^35^ and that SSC correlates better with sorbitol than with sugars. Sorbitol represents less than 10% of the total sugar in apples (Table 1) and its sweetening index is lower than sucrose, fructose and glucose^36^. Therefore, the amount of sorbitol alone cannot explain sweetness. Sorbitol content in apple is most likely related to the ripening stage of the fruit and is positively correlated with SSC, glucose, fructose and total sugar content thus it may reflect behaviours of other powerful components which are not dealt with in this study. As a result, we must take into account the interactions between metabolites rather than the amount of a single component to explain apple sweetness or sensory perception in general.

The measured analytical variables (sucrose, fructose, glucose, xylose, sorbitol, malic acid, SSC) were used to build a multiple regression model to predict apple sweetness (model 1). Because the variables are highly correlated with each other, we used the OPLS (Orthogonal Projections to Latent Structures) regression to build the prediction model. Sucrose, fructose, glucose, xylose, sorbitol, malic acid and SSC were used as an X variable matrix to predict sweetness as a y output variable.

**Figure 1:**
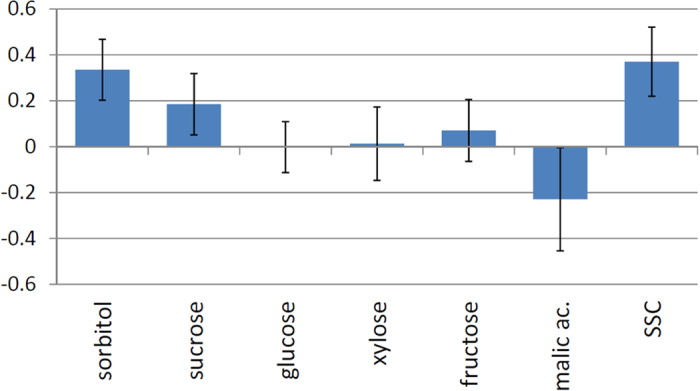
Coefficients of OPLS regression model for sweetness referring to scaled and centered data. Confidence intervals (95%) are derived from jack knifing.

The cross-validated model (leave-one-out) explains 59.3% of the perceived sweetness of apples and its prediction ability is (Q^2^) 49.3%. According to the model coefficients, SSC, sorbitol and sucrose contributed positively to sweetness, whereas malic acid contributed negatively (Fig. 1). Glucose, xylose and fructose do not significantly contribute to the perceived sweetness in apples. Excluding them from the model (model 2) increases the prediction ability (Q^2^) slightly, to 52.3%.

The model reveals that taking into account interactions between variables increases our ability to explain apple sweetness from 44 (for simple correlation between sorbitol and sweetness) to 59%. Although most significant, sugars, sorbitol, soluble solids and malic acid are not the only factors responsible for the perception of apple sweetness.

### Volatile compounds in apple headspace

From the headspace of analysed apples, 95 peaks were considered, and semi-quantitative data were recorded (Supplementary Table 1). Most of the peaks have been identified. More than half are esters (40 compounds) or alcohols (19 compounds). On average, the most abundant compounds (with a relative amount higher than 2%) are: hexyl acetate (24.4% of total amount of volatile compounds) ranging from 0.93 to 267.65 μg kg^−1^; hexanol (23.0% of total amount of volatile compounds) ranging from 15.76 to 169.23 μg kg^−1^; butyl acetate (15.4% of total amount of volatile compounds) ranging from 1.05 to 137.36 μg kg^−1^; 3-methylbutyl acetate (13.5% of total amount of volatile compounds) ranging from 0.86 to 101.87 μg kg^−1^; butanol (4.3% of total amount of volatile compounds) ranging from 0.88 to 50.17 μg kg^−1^; and 2-methyl-1-butanol (3.4% of total amount of volatile compounds) ranging from 2.41 to 37.12 μg kg^−1^. These results are consistent with previous studies^1^,^37^. This dataset was used for the analyses reported below.

### Effect of volatile compounds on perceived sweetness

Due to the low correlation between sugar content and sweetness and the fact that a predictive model including sugars, sorbitol, malic acid and SSC explains less than 60% of perceived sweetness, we decided to investigate the possible role of volatile compounds on the perception of sweetness.

Table 4 reports a list of volatile compounds that significantly correlate with sweetness. Of the 16 significant compounds reported (p < 0.05), 13 are positively correlated to sweetness and 3 negatively. The major group of compounds positively correlated to sweetness are esters (7 out of 16). Propyl esters showed the highest correlation coefficients: 0.652, 0.555 and 0.552 for propyl 2-methylbutanoate, propyl hexanoate and propyl propanoate, respectively. The other positively correlated compounds are the three isomers of farnesene, 2-methyl-1-butanol, benzothiazole and 5-ethyldihydro-2(3 H)-furanone. The negatively correlated compounds are 1-octen-3-one, 2-heptenal and 1-octen-3-ol.

**Figure 2:**
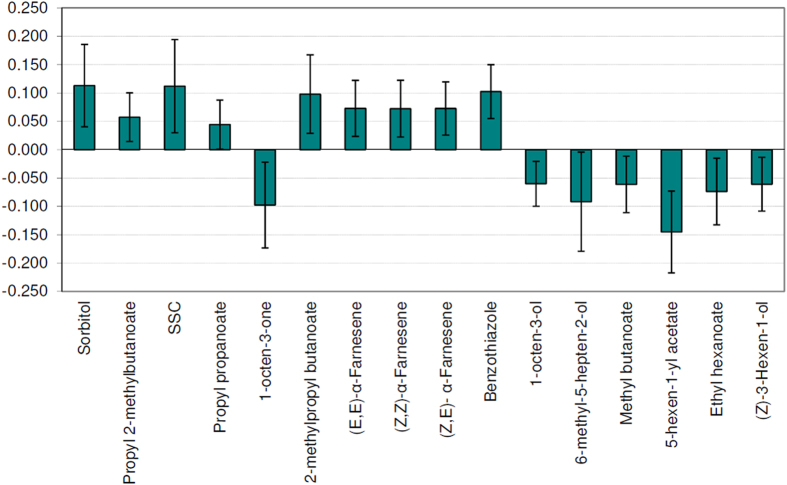
Significant regression coefficients with jack knifing intervals from the OPLS regression model for sweetness listed in order of variable importance in projection (VIPs) >0.95.

The volatile compounds (95 variables) together with single sugars (sucrose, glucose, fructose, and xylose), sorbitol, malic acid and SSC were included in a new OPLS model (model 3) to predict the sweetness perceived by the sensory panel. The new model explains 92.0% of sample sweetness variance with a predictive ability (Q^2^) of 62.7% (leave-one-out). The Supplementary Table 2 shows at glance model information for each model discussed in the manuscript. For the model 3, the variable importance in projection scores (VIPs) and correlation coefficients were calculated. After the validation procedure, based on jack knifing (95%), the coefficients with VIPs higher than 0.95 resulted in 16 variables, as depicted in Fig. 2. The results of the OPLS model are congruent with univariate analysis and the compounds correlating with sweetness (reported in Table 4) are confirmed by PLS model. Among the non-volatile compounds, those contributing to the model are sorbitol and SSC, in agreement with correlation coefficients: these variables correlated to sweetness better than total or single sugars. The contribution of malic acid and sucrose to this new model was negligible. The volatile compounds with a positive contribution to the model are three esters, the three farnesene isomers, and benzothiazole. Farnesene and esters are developed during apple ripening^38^, when the conversion of starch to single sugars occurs^25^,^39^,^40^. Furthermore, esters strongly contribute to sweet fruity descriptors in apple^41^,^42^ that may elicit odour-induced enhancement of sweetness perception^20^. Tanaka and colleagues found a positive correlation between the intensities of sweetness with floral and fruity attributes and negative correlation with the green attributes in fuji apples^43^.

The other seven compounds, 1-octen-3-one, 1-octen-3-ol, 6-methyl-5-hepten-2-ol, methyl butanoate, 5-hexenyl acetate, ethyl hexanoate and cis-3-hexen-1-ol, negatively contribute to the sweetness model. The odours associated with these compounds are not “sweet-congruent”: earthy-fungal for 1-octen-3-one and 1-octen-3-ol; and green-herbaceous for 5-hexenyl acetate, 6-methyl-5-hepten-2-ol and (Z)-3-hexen-1-ol. Ethyl hexanoate and methyl butanoate can elicit fruity-pineapple and fruity-apple or fruity-banana, respectively, at low concentration or green-banana and pungent or ethereal at higher concentrations^44^.

According to the developed model, volatile compounds in apples can explain approximately 33% of perceived sweetness, demonstrating once again that sensory perception is regulated by multisensory stimuli.

Former studies have indicated a possible role for volatile compounds in enhancing or contrasting the sweetness in other vegetal matrices. For example, Baldwin and co-workers found a negative correlation between some volatile compounds related to green notes (hexanal, (E)-2-heptenal, (Z)-3-hexenol, (Z)-3-hexenal) and sweetness in tomato^22^. In strawberries, six compounds (1-penten-3-one, γ-dodecalactone, pentyl butanoate, hexyl butanoate, hexyl acetate, 2-pentyl butanoate) were found to significantly enhance sweetness intensity independently of all three sugars (glucose, fructose, or sucrose) quantified^23^.

## Conclusion

For the first time, the role of the major sugars and sorbitol in contributing to sweetness perception was investigated in apples, showing no direct interaction between the ratios of different sugars and sweetness. We developed an improved model (62.7%) to predict perceived sweetness in apple that includes not only soluble solid components (sugars and acids) but also volatile compounds.

When sensory characteristics are studied, it is essential to consider, in a multivariate fashion, all chemical and physical parameters involved rather than using a single stimulus. Only in this way it is possible to try to describe a complex fruit trait such as sweetness.

The role of sensory analysis, using trained judges in combination with instrumental determinations and/or consumer groups, remains central to the description and quantification of complex fruit traits such as sweetness and its influence on consumer preference.

In conclusion, the search for increasing sweetness in apple breeding programmes must take into account not only sugar content but also factors such as volatile compounds, texture parameters, minor components (i.e., polyphenols) and information from sensory panels.

## Methods

### Apple

Forty apple batches were collected and evaluated during the 2013 harvesting season (Table 2). Collected apples belonged to 17 different cultivars/accessions. Some batches contained the same cultivar, with fruits coming from different orchards in different locations (information not shown). For all batches, fruit were picked at the commercial harvest time typical of each variety and stored for 2 months at standard refrigeration conditions (2 °C, 98% RH, normal atmosphere). Prior to analysis, fruits were kept at room temperature for 24 h. Twenty fruits per batch were chosen by controlling weight and a non- destructive measurement of ripening degree (DA-meter, Turoni s.r.l., Forlì, Italy) to maximize homogeneity (the selected fruits ranged in the interval of mean ± σ/2 for both parameters). To prepare samples for sensory evaluation, three horizontal sections 1.2 cm high, were cut around the equatorial plane perpendicular to the core of the fruit. The slices were immediately dipped into an antioxidant solution (0.2% citric acid, 0.2% ascorbic acid, 0.5% calcium chloride) for 30 s. Cylindrical shapes (1.8 cm diameter, 5 or 6 cylinders per slice) were cut from the flesh using a commercial apple corer (Tescoma, Brescia, Italy). Flesh cylinders from the same apple were used for sensory and instrumental analyses.

### Sensory data

Sweetness and sourness were evaluated by a trained panel of 19 assessors with several years of experience in apple quantitative descriptive analysis based on a consensus-developed lexicon^13^. The intensity of each attribute was scored by the panel on a 100-mm linear scale with three anchored points: 0 (minimum intensity or absence), 100 (maximum intensity) and 50 (an intermediate level). The references provided to the panellists during the training were 20 g kg^−1^ and 80 g kg^−1^ fructose aqueous solutions for the minimum and maximum intensity for sweetness and 0.6 g kg^−1^ and 2.0 g kg^−1^ of citric acid aqueous solutions for the minimum and maximum intensity for sourness. Sensory analyses were performed in individual computerized booths in the Fondazione Edmund Mach Sensory Laboratory equipped with FIZZ 2.46 A software (Biosystemes, Couternon, France) under red light to avoid any visual influence of apple flesh colour on the evaluations. Apple cylinders (see section 4.1) were presented to each panellist in clear plastic cups (eight cylinders per cup) and encoded with a three-digit random code following a Williams Latin square design.

### Chemical analyses

#### Sample preparation

A purée from 50 g of apple flesh cylinders (see section 4.1) and distilled water (ratio 1:1) was prepared in a blender. The purée was transferred to sealed bottles and stored at −20 °C until the analysis. The thawed purée was centrifuged at 12,000 g for 15 min at 4 °C. The supernatant was filtered through a 0.45 μm Millipore membrane. The collected filtrate served as the sample solution used to determine sugar and malic acid content.

#### Sugars and sorbitol

Sugars (sucrose, glucose, fructose, xylose) and sorbitol were analysed using high-pressure capillary ion chromatography (Dionex ICS-5000, Thermo Fisher Scientific, Waltham, MA, USA) with pulsed amperometric detection (HPIC-PAD) by injecting 5 μL of sample solution onto a Dionex UltiMate 3000 (Thermo Fisher Scientific, Waltham, MA, USA) equipped with a Dionex CarboPac PA200 column (3 × 250 mm). A 3 steps isocratic solvent system consisting of A (NaOH, 1 M), B (deionized water) and C (NaOH, 30 mM) was used as the mobile phase. The 3 isocratic steps were as follows: initial conditions of B (97%) + C (3%) for 13 min; then A (10%) + B (90%) for 3 min; and B (97%) + C (3%) for 5 min. The total run time was 21 min. The flow rate was 0.3 ml min^−1^ except for the interval between 9.5 and 19 min, where the flow rate was 0.4 ml min^−1^.

Stock solutions of fructose, sorbitol, xylose, glucose and sucrose were prepared individually in deionized water and working solutions for calibrations were prepared using appropriate dilutions of these. The coefficient of variation for fructose, glucose, xylose, sorbitol was below 2%, whereas for sucrose, it was approximately 4%. Total sugar content was calculated as the sum of fructose, xylose, glucose and sucrose.

#### Soluble solid content

The concentration of soluble solid content (% SSC) was measured in the combined juice extracted by mechanical compression of 12 cylinders (see sample selection), each cylinder collected from a different fruit. The measures were performed in two replicates using a DBR35 refractometer (XS Instruments, Poncarale, Brescia, Italy) and are expressed here as °Brix.

#### Organic acids

Organic acid determination (malic and ascorbic) was performed by adapting the procedure described by Chen and co-workers^45^.

A ThermoScientific Ultimate 3000RS (Thermo Fisher Scientific, Waltham, MA, USA) Ultra High Performance Liquid Chromatography unit coupled with an electrospray ionisation (ESI) Q Exactive™ Hybrid Quadrupole-Orbitrap Mass Spectrometer (Thermo Fisher Scientific, Waltham, MA, USA) was used to quantify the malic acid in apple extracts by injecting 20 μL of sample solution. Separation of malic and ascorbic acids was performed with an analytical column (Rezex ROA Organic acid H^+^; 30 cm × 7.8 mm, 8 μn, Phenomenex, Torrance, CA). The mobile phase (water with 0.5% of formic acid) flow-rate was 0.8 mL min^−1^ and the column temperature was 50 °C. The instrument was operated in the negative ionisation mode. The operating conditions for ESI were as follows: sheath gas flow rate of 40 arbitrary units; sweep gas flow rate of 0 arbitrary units; capillary voltage 3,500 V; capillary temperature 350 °C and gas temperature 300 °C. Data were collected using full scans (scan range 100–1500 m/z) and the ions at m/z 133.01425 and 175.02481 were used to quantify malic and ascorbic acid, respectively, expressed as g of organic acid per kg of apple flesh.

### GC-MS analysis of volatile compounds

Samples for the analysis of volatile compounds were prepared following the procedure described in a previous work^46^, starting from the apple flesh cylinders as described in the section 4.1. Before analysis, the samples introduced into 22 ml vials were spiked with 2-octanol for semi-quantitative purposes. After 10 min of equilibration at 40 °C, headspace volatile compounds were extracted and concentrated on a 2-cm solid-phase microextraction fibre coated with divinylbenzene/carboxen/polydimethylsiloxane (50/30 μm, DBV/CAR/PDMS, Supelco, Bellefonte, PA, USA) for 30 min. Volatile compounds adsorbed on the SPME fibre were desorbed at 250 °C in the injector port of a GC interfaced with a mass detector in electron impact ionisation mode (EI, internal ionisation source; 70 eV) with a scan range from m/z 35 to 300 (GC Clarus 500, PerkinElmer, Norwalk CT, USA). Separation was achieved on a HP-Innowax fused-silica capillary column (30 m, 0.32 mm ID, 0.5 μm film thickness; Agilent Technologies, Palo Alto, CA, USA) by applying the following GC oven temperature program: 40 °C for 3 min, then 40–220 °C at 4 °C min^−1^, 220 °C for 1 min, then 220–250 at 10 °C min^−1^, 250 °C for 1 min. Helium was used as the carrier gas, with a constant column flow rate of 1.5 mL min^−1^. Peak identity was based on mass spectra matching with the NIST Standard Reference Database (NIST2014) and comparison of linear retention indices (LRI) with literature, as well as from authentic reference standards when available. A series of n-alkanes (C7–C30 from Supelco) was injected under the same chromatographic conditions for the calculation of LRIs. All reference compounds were purchased from Sigma-Aldrich (Sigma, Aldrich, and Fluka catalogues).

### Statistical analyses

Descriptive statistics, correlation and multiple regression analysis were performed using Statistica 9.1 software (StatSoft, Inc., Tulsa, OK). Regression models were developed using the Orthogonal Projections to Latent Structures (OPLS) procedure^47^. SIMCA-P + 12.0 (Umetrics, Umeå, Sweden) was used to build and validate the predictive models^48^. Variables were centred and scaled to the unit variance before model development. The OPLS model was validated using a cross-validation procedure as previously reported^49^.

## Additional Information

**How to cite this article**: Aprea, E. *et al*. Sweet taste in apple: the role of sorbitol, individual sugars, organic acids and volatile compounds. *Sci. Rep.* **7**, 44950; doi: 10.1038/srep44950 (2017).

**Publisher's note:** Springer Nature remains neutral with regard to jurisdictional claims in published maps and institutional affiliations.

## Supplementary Material

Supplementary Tables

## Figures and Tables

**Table 1:** Apple composition (mean values): individual sugars, sorbitol, malic acid, soluble solid content and total amount of sugars.

Batch ID^a^	Sucrose	Fructose	Glucose	Xylose	Sorbitol	Malic acid	Soluble Solids	Total sugars^b^
	g kg^−1^	g kg^−1^	g kg^−1^	g kg^−1^	g kg^−1^	g kg^−1^	°Brix	g kg^−1^
*1*	47.1	51.3	29.4	1.5	9.2	12.9	14.9	129.3
*2*	35.0	37.4	20.8	0.5	6.0	19.4	14.9	93.7
*3*	39.8	59.2	27.7	0.8	12.7	27.3	14.9	127.5
*4*	42.0	52.1	28.6	0.9	12.6	18.6	14.9	123.5
*5*	51.4	48.6	24.2	1.3	10.7	18.5	13.0	125.4
*6*	43.8	55.9	26.8	0.9	8.5	15.1	14.9	127.3
*7*	75.2	42.0	14.8	0.5	7.1	48.4	14.6	132.5
*8*	84.2	39.8	13.7	1.4	7.3	23.7	13.5	139.2
*9*	44.8	38.8	24.8	1.2	3.7	19.1	13.7	109.6
*10*	50.8	56.3	20.8	0.6	7.8	19.2	13.5	128.4
*11*	56.1	44.6	25.1	1.8	4.3	17.5	13.7	127.5
*12*	64.8	44.7	26.2	1.7	12.9	13.8	15.8	137.4
*13*	62.9	47.7	24.7	1.4	12.4	19.2	15.8	136.8
*14*	62.3	41.4	13.2	1.1	7.0	29.4	14.0	117.9
*15*	45.7	56.5	19.9	1.1	2.0	40.9	—	123.1
*16*	45.9	50.1	30.2	1.2	9.4	16.5	13.0	127.4
*17*	74.7	42.9	11.1	0.5	6.4	56.3	13.8	129.2
*18*	28.5	27.0	18.5	0.9	1.9	20.7	13.7	74.7
*19*	91.0	37.3	14.2	0.4	3.4	32.8	13.4	142.9
*20*	51.2	54.9	24.7	1.3	7.0	29.3	13.4	132.1
*21*	22.3	36.1	17.6	1.0	2.1	18.0	12.6	77.0
*22*	35.6	53.2	25.6	1.4	5.1	18.2	12.9	115.9
*23*	41.4	35.7	14.9	0.4	4.5	29.2	13.3	92.4
*24*	58.7	51.4	19.7	0.6	6.2	32.6	13.3	130.4
*25*	38.5	61.0	25.4	1.3	3.6	16.7	12.6	126.2
*26*	63.6	44.2	14.0	0.7	3.5	34.6	—	122.5
*27*	82.1	35.0	12.0	0.6	1.5	30.3	14.9	129.6
*28*	35.1	46.0	17.6	0.8	2.5	26.7	10.5	99.5
*29*	39.0	51.7	18.9	0.8	2.6	26.3	10.5	110.3
*30*	53.3	57.5	16.0	0.5	4.7	30.0	9.5	127.3
*31*	75.5	32.0	13.5	0.3	2.0	29.6	14.1	121.3
*32*	54.2	42.0	27.5	0.5	5.7	39.2	10.6	124.1
*33*	22.2	46.9	26.2	1.5	1.8	21.8	12.6	96.8
*34*	22.6	44.8	25.8	1.3	1.3	16.3	12.6	94.4
*35*	42.7	42.9	19.1	0.9	3.0	31.0	12.8	105.6
*36*	42.9	39.3	18.6	0.8	2.3	37.3	12.8	101.6
*37*	38.9	39.2	17.6	0.8	2.5	23.4	12.8	96.4
*38*	47.7	37.5	23.9	0.0	3.3	33.8	10.3	109.0
*39*	28.1	35.8	22.7	0.9	4.1	78.6	13.2	87.4
*40*	46.5	51.8	19.2	1.0	6.5	43.9	11.7	118.5

^a^See Table 2 for batch details; ^b^Sum of sucrose, fructose, glucose, xylose.

**Table 2:** Sweetness and sourness (mean values) of apples as scored by the trained sensory panel on a 0–100 linear scale.

Batch ID	Sweetness	Sourness	cultivar/accession
	(0–100)	(0–100)	
*1*	69.2	16.4	Fuji
*2*	69.1	16.3	Fuji
*3*	67.5	15.0	Fuji
*4*	67.4	14.0	Fuji
*5*	63.7	16.3	Fujion
*6*	63.7	15.9	Fuji
*7*	62.8	26.8	Dalinette
*8*	60.8	21.4	Smeralda
*9*	58.6	28.1	Opal
*10*	58.5	19.1	FEM_5*
*11*	57.4	19.6	Opal
*12*	57.2	28.2	Fujion
*13*	57.1	28.1	Fujion
*14*	54.1	27.1	Dalinette
*15*	53.7	30.5	Golden Delicious
*16*	52.3	23.5	Fujion
*17*	52.0	35.1	Dalinette
*18*	52.0	21.7	Opal
*19*	51.0	42.3	Crimson Crisp
*20*	50.6	17.8	Modi
*21*	50.2	29.3	Golden Delicious
*22*	47.5	18.1	FEM_11*
*23*	46.8	49.6	Opal
*24*	46.7	49.5	Opal
*25*	46.4	24.6	Golden Delicious
*26*	45.2	58.1	Opal
*27*	44.6	61.4	Crimson Crisp
*28*	43.6	9.7	Morgendurf
*29*	43.5	9.6	Morgendurf
*30*	43.2	46.7	Pinova Roho
*31*	42.4	48.6	Crimson Crisp
*32*	41.6	38.2	Doriane
*33*	36.7	30.8	Golden Delicious
*34*	36.7	30.7	Golden Delicious
*35*	34.9	54.4	Braeburn
*36*	32.3	48.2	Braeburn
*37*	30.9	66.7	Braeburn
*38*	30.1	73.4	Granny Smith
*39*	29.4	71.8	Renetta Canada grey
*40*	27.3	62.2	Renette Canada

*Apple genotypes of FEM breeding programme under evaluation.

**Table 3:** Pearson’s correlation coefficients (r) between perceived sweetness and instrumental measurements of SSC, individual sugars, sorbitol and malic acid.

	SSC	sorbitol	sucrose	glucose	Xylose	fructose	malic ac.	sugars	acids/sugars	sweetness
SSC	1	**0.514**	0.282	0.106	0.303	−0.086	−0.205	0.269	−0.226	**0.635**
sorbitol	**0.514**	1	0.222	**0.437**	0.301	**0.486**	−0.189	**0.578**	−0.303	**0.661**
sucrose	0.282	0.222	1	**−0.482**	−0.186	−0.141	0.180	**0.771**	−0.101	0.237
glucose	0.106	**0.437**	**−0.482**	1	**0.513**	**0.496**	**−0.402**	0.064	−0.317	0.221
xylose	0.303	0.301	−0.186	**0.513**	1	0.308	**−0.455**	0.117	**−0.381**	0.249
fructose	−0.086	**0.486**	−0.141	**0.496**	0.308	1	−0.268	**0.478**	**−0.379**	0.234
malic ac.	−0.205	−0.189	0.180	**−0.402**	−0.455	−0.268	1	−0.071	**0.935**	**−0.449**
Total sugars	0.269	**0.578**	**0.771**	0.064	0.117	**0.478**	−0.071	1	**−0.373**	**0.410**
acids/sugars	−0.226	−0.303	−0.101	−0.317	**−0.381**	**−0.379**	**0.935**	**−0.373**	1	**−0.521**
sweetness	**0.635**	**0.661**	0.237	0.221	0.249	0.234	**−0.449**	**0.410**	**−0.521**	1

Correlation with a p < 0.05 are marked in bold.

**Table 4:** Pearson’s correlation coefficients between volatile compounds and perceived sweetness in apple.

Volatile compounds	r	P
Propyl 2-methylbutanoate	0.652	<0.001
Propyl hexanoate	0.555	<0.001
Propyl propanoate	0.552	<0.001
2-Methyl-1-butanol	0.528	0.001
3-Methylbutyl acetate	0.51	0.001
3-Methylbutyl butanoate	0.486	0.002
2-Methylpropyl butanoate	0.486	0.002
(Z, Z)-alpha Farnesene	0.471	0.003
(E, E)-alpha Farnesene	0.47	0.003
(Z, E)-alpha Farnesene	0.451	0.005
Benzothiazole	0.406	0.012
Propyl acetate	0.399	0.013
5-Ethyldihydro-2(3H)-furanone	0.336	0.039
1-Octen-3-ol	−0.37	0.022
2-Heptenal	−0.404	0.012
1-Octen-3-one	−0.491	0.002

Only compounds with a p < 0.05 are reported.
