# Supplementary material for: Sweet taste in apple: the role of sorbitol, individual sugars, organic acids and volatile compounds
Source: Sci Rep. 2017 Mar 21;7:44950. doi: 10.1038/srep44950 (PMC5359574; doi:10.1038/srep44950)
Supplement: Supplementary Tables [file srep44950-s1.doc]

## Supplementary information

**Sweet taste in apple: the role of sorbitol, individual sugars, organic acids and volatile compounds**

Eugenio Aprea, Mathilde Charles, Isabella Endrizzi, Maria Laura Corollaro^1^, Emanuela Betta, Franco Biasioli, Flavia Gasperi

**Contents:** Supplementary Table 1

Supplementary Table 2

**Suppl. Table 1.** Volatile compounds in the apple headspace ordered according to elution order on a polar column

|  | **Compound** | **RT** (min) | **LRI^a^** | **ID^b^** |
| --- | --- | --- | --- | --- |
| 1 | Butanal | 2.94 | 880 | S, L, M |
| 2 | Ethyl acetate | 3.10 | 899 | S, L, M |
| 3 | Propyl acetate | 4.82 | 982 | L, M |
| 4 | Methyl butanoate | 5.11 | 995 | L, M |
| 5 | Methyl 2-methylbutanoate | 5.67 | 1019 | L, M |
| 6 | Isobutyl acetate | 5.76 | 1023 | L, M |
| 7 | Ethyl butanoate | 6.43 | 1049 | L, M |
| 8 | Propyl propanoate | 6.66 | 1058 | L, M |
| 9 | Ethyl 2-methylbutanoate | 6.89 | 1066 | L, M |
| 10 | Butyl acetate | 7.45 | 1090 | L, M |
| 11 | Hexanal | 7.73 | 1101 | S, L, M |
| 12 | 2-Methyl-1-propanol | 8.11 | 1112 | L, M |
| 13 | 3-Methylbutyl acetate (Isoamyl acetate) | 9.01 | 1141 | L, M |
| 14 | Ethyl pentanoate (Ethyl valerate) | 9.49 | 1142 | L, M |
| 15 | Propyl 2-methylbutanoate | 9.58 | 1158 | M |
| 16 | Butyl propanoate | 9.66 | 1161 | S, L, M |
| 17 | 1-Butanol | 9.79 | 1166 | L, M |
| 18 | 2-Methylpropyl butanoate | 10.28 | 1179 | S, L, M |
| 19 | Pentyl acetate (Amyl acetate) | 10.77 | 1196 | S, L, M |
| 20 | Methyl hexanoate | 11.25 | 1202 | L, M |
| 21 | Limonene | 11.54 | 1214 | L, M |
| 22 | 2-Methyl-1-butanol | 11.87 | 1228 | L, M |
| 23 | (E)-2-Hexenal | 12.28 | 1238 | S, L, M |
| 24 | Butyl butanoate | 12.29 | 1239 | L, M |
| 25 | Butyl 2-methylbutanoate | 12.72 | 1252 | S, M |
| 26 | Ethyl hexanoate | 12.83 | 1254 | L, M |
| 27 | 3-methyl-2-butenyl acetate (Prenyl acetate) | 13.34 | 1256 | M |
| 28 | Pentanol | 13.38 | 1269 | L, M |
| 29 | 3-Methylbutyl butanoate (Isoamyl butanoate) | 13.89 | 1283 | S, L, M |
| 30 | Hexyl acetate | 14.19 | 1291 | S, L, M |
| 31 | 1-Octen-3-one | 15.15 | 1319 | S, L, M |
| 32 | (Z)-3-Hexenyl acetate | 15.41 | 1335 | S, L, M |
| 33 | Pentyl butanoate (Amyl butanoate ) | 15.66 | 1336 | S, L, M |
| 34 | (E)-3-Hexenyl acetate | 15.69 | 1336 | M |
| 35 | Propyl Hexanoate | 15.71 | 1336 | L, M |
| 36 | (E)-2-Heptenal | 15.90 | 1337 | L, M |
| 37 | 5-Hexenyl acetate | 16.01 | 1338 | M |
| 38 | (E)-2-Hexenyl acetate | 16.27 | 1352 | L, M |
| 39 | 6-Methyl-5-hepten-2-one | 16.35 | 1354 | L, M |
| 40 | Hexyl 2-methylpropanoate (Hexyl Isobutanoate) | 16.49 | 1355 | M |
| 41 | Hexanol | 16.88 | 1368 | S, L, M |
| 42 | Heptyl acetate | 17.59 | 1384 | M |
| 43 | (Z)-3-Hexenol | 17.90 | 1399 | S, L, M |
| 44 | Nonanal | 18.24 | 1402 | L, M |
| 45 | (E)-2-Hexenol | 18.62 | 1421 | S, L, M |
| 46 | (Z)-5-Hexen-1-ol | 18.70 | 1426 | S, L, M |
| 47 | Butyl hexanoate | 18.85 | 1428 | L, M |
| 48 | Hexyl butanoate | 18.94 | 1431 | S, L, M |
| 49 | Unidentified 1 | 19.20 | 1440 |  |
| 50 | 2-Methylhexyl butanoate | 19.28 | 1441 | M |
| 51 | (E)-Linalool oxide A | 19.72 | 1455 | L, M |
| 52 | 1-Octen-3-ol | 20.04 | 1465 | L, M |
| 53 | 1-Heptanol | 20.20 | 1470 | L, M |
| 54 | 6-Methyl-5-hepten-2-ol | 20.45 | 1478 | L, M |
| 55 | Furfural | 20.55 | 1480 | L, M |
| 56 | (Z)-Linalool oxide B | 20.63 | 1482 | L, M |
| 57 | 2-Ethyl-1-hexanol | 21.31 | 1505 | L, M |
| 58 | Decanal | 21.61 | 1513 | L, M |
| 59 | Heptyl butanoate | 22.14 | 1530 | M |
| 60 | Benzaldehyde | 22.40 | 1537 | S, L, M |
| 61 | Furfuryl acetate | 22.83 | 1547 | L, M |
| 62 | Linalool | 23.08 | 1559 | L, M |
| 63 | Octanol | 23.41 | 1570 | S, L, M |
| 64 | Hexyl hexanoate | 24.99 | 1620 | L, M |
| 65 | γ-Butyrolactone | 25.48 | 1637 | L, M |
| 66 | Acetophenone | 26.18 | 1660 | L, M |
| 67 | Nonanol | 26.46 | 1671 | S, L, M |
| 68 | Unidentified 2 | 26.74 | 1680 |  |
| 69 | p-Allyl anisole (Estragole) | 26.78 | 1681 | L, M |
| 70 | α-Terpineol | 27.48 | 1705 | L, M |
| 71 | 5-Ethyldihydro-2(3H)-furanone (γ-Caprolactone) | 27.61 | 1710 | S, L, M |
| 72 | 3-Hydroxy butyl butanoate | 27.72 | 1714 | M |
| 73 | Tetradecanal (Myristaldehyde) | 27.91 | 1721 | M |
| 74 | 3-(Methylthio)-1-propanol (Methionol) | 28.04 | 1726 | S, L, M |
| 75 | (Z,E)-α-Farnesene | 28.36 | 1736 | M |
| 76 | Benzyl acetate | 28.44 | 1740 | L, M |
| 77 | Unidentified 3 | 28.62 | 1746 |  |
| 78 | (E,E)-α-Farnesene | 28.99 | 1760 | S, L, M |
| 79 | 1-Decanol | 29.36 | 1775 | L, M |
| 80 | (Z,Z)-α-Farnesene | 30.03 | 1799 | M |
| 81 | 3-Decenol | 30.27 | 1808 | M |
| 82 | 2,4-Dimethyl benzaldehyde | 30.73 | 1824 | M |
| 83 | Phenethyl acetate | 30.81 | 1827 | L, M |
| 84 | β-Damascenone | 30.94 | 1831 | L, M |
| 85 | Anethol (Isoestragole) | 31.21 | 1839 | S, L, M |
| 86 | Geranyl acetone | 31.84 | 1865 | L, M |
| 87 | 2-Phenyl ethanol (Benzenethanol) | 33.30 | 1921 | L, M |
| 88 | Benzothiazole | 34.41 | 1963 | L, M |
| 89 | Dodecanol | 34.79 | 1973 | L, M |
| 90 | Phenol | 35.77 | 2000 | L, M |
| 91 | Hexanoic acid | 37.10 | 2036 | M |
| 92 | 4-Methyl phenol (p-Cresol) | 37.82 | 2056 | M |
| 93 | 2-Phenoxy ethanol (Rose ether) | 39.00 | 2150 | S, L, M |
| 94 | Octanoic acid | 42.17 | 2178 | M |
| 95 | Nonanoic acid | 44.50 | 2228 | S, L, M |

^a^ calculated linear retention index;

^b^ Identification by means injection of pure standard compound (S), matching of LRI with literature data (L) or mass spectra matching with MS library (M)

**Suppl. Table 2.** Information for “Sweetness” predicting models

| N° | Type of model | N. of predicting variables | Type of variables ^a^ | % of explained variance (R^2^) | % of predicted sweetness (Q^2^) | RSMECV ^b^ |
| --- | --- | --- | --- | --- | --- | --- |
| 1 | OPLS | 7 | SCa | 59.3 | 49.3 | 8.031 |
| 2 | OPLS | 4 | SCb | 59.3 | 52.3 | 7.927 |
| 3 | OPLS | 102 | VC | 92.0 | 62.7 | 7.029 |

^a^ SCa: soluble component comprising SSC, sucrose, fructose, glucose, xylose, sorbitol and malic acid; SCb: soluble component comprising SSC, sucrose, sorbitol and malic acid; VC: volatile compounds

^b^ RSMECV: root square mean error using leave-one-out cross validation scheme
